# Effects of a Nanotechnology-Based Application on Balance Control in Hearing Aid Users

**DOI:** 10.3390/audiolres16020042

**Published:** 2026-03-08

**Authors:** Francesca Campoli, Andrea Fabris, Donatella Di Corrado, Dorota Kostrzewa-Nowak, Robert Nowak, Lucio Caprioli, Vincenzo Cristian Francavilla, Elvira Padua, Giuseppe Messina

**Affiliations:** 1 Department of Human Sciences and Promotion of the Quality of Life, San Raffaele University, 00166 Rome, Italy; francesca.campoli@uniroma5.it (F.C.); andrea.fabris@uniroma5.it (A.F.); elvira.padua@uniroma5.it (E.P.); 2 Sports Engineering Lab, Department Industrial Engineering, University Rome Tor Vergata, 00133 Rome, Italy; lucio.caprioli@uniroma2.it; 3 Department of Sport Sciences, Kore University, Cittadella Universitaria, 94100 Enna, Italy; donatella.dicorrado@unikore.it; 4 Department of Clinical and Molecular Biochemistry, Pomeranian Medical University in Szczecin, 70-204 Szczecin, Poland; dorota.kostrzewa.nowak@pum.edu.pl; 5 Institute of Physical Culture Sciences, University of Szczecin, 70-453 Szczecin, Poland; robert.nowak@usz.edu.pl; 6 Department of Pathology, Pomeranian Medical University in Szczecin, 70-204 Szczecin, Poland; 7 Department of Medicine and Surgery, Kore University, 94100 Enna, Italy; vincenzo.francavilla@unikore.it; 8 PLAB Research Institute, 90134 Palermo, Italy

**Keywords:** posture, postural balance, nanotechnological device, hearing aids, balance disorders

## Abstract

Background: Balance impairment and falls are a major health concern in older adults. Beyond vestibular and visual factors, growing evidence indicates that age-related hearing loss contributes to postural instability through altered multisensory integration. However, interventions addressing the interaction between auditory input and postural control remain limited. This study examined whether integrating Taopatch^®^ nanotechnology, based on localized photobiomodulation, into conventional hearing aids could influence postural control in individuals with hearing loss. Methods: Forty experienced hearing aid users (mean age 77.3 ± 15.6 years) completed five postural assessments using a SensorMedica^®^ baropodometric platform. Four sessions employed a placebo patch identical in appearance to the active device, and the fifth used Taopatch^®^. Static and stabilometric parameters were analyzed under open- and closed-eye conditions. Results: Significant improvements were observed with the Taopatch^®^-integrated device. Sway path length (−8%, *p* = 0.002), mean velocity (−8%, *p* = 0.002), and low-frequency sway (−30%, *p* = 0.04) decreased, indicating smoother and more efficient postural control. A lateral redistribution of plantar load and an increase in contact surface area (up to +15%) were also found. These effects were less evident without visual input. Conclusions: Preliminary findings suggest that localized photobiomodulation integrated into hearing aids may positively influence postural stability in older adults with hearing impairment, possibly by supporting sensory integration processes. Further controlled studies are needed to confirm these effects and clarify the underlying mechanisms.

## 1. Introduction

Postural control relies on the integration of vestibular, visual, and somatosensory inputs, but the auditory and balance systems are also closely interconnected, both anatomically and functionally. This proximity enables auditory input to serve as a crucial reference for spatial orientation, motor coordination, and postural stability, integrating with visual and somatosensory cues [1].

Several studies confirm that auditory stimuli influence postural control, serving as external references that can enhance body stability [2,3]. Accordingly, hearing loss is increasingly recognized as a factor associated with diminished balance. Individuals with moderate hearing impairment have shown significantly impaired postural control compared to normal-hearing counterparts [4], and hearing loss is a known risk factor for falls in older adults.

Lin and Ferrucci [5] reported that even mild hearing loss triples the risk of falling, while more recent analyses further support the association between auditory deficits, impaired postural control, and increased fall incidence [4]. The risk of falls in older people also has a significant impact on quality of life, both socially and economically. Hospitalisations due to falls account for approximately 32.9% of the total annually [6]. Moreover, such falls lead to functional limitations with individuals reporting problems with mobility, activities of daily living, and anxiety/depression [7].

Age-related sensory decline can reduce stability, but this may be partially compensated for by enhancing sensory input through visual and auditory cues [8]. Hearing aids may partially compensate for this deficit. Rumalla [9] observed improvements in postural stability among hearing aid users, suggesting that sound amplification may help restore sensory integration and spatial awareness. These observations are consistent with earlier studies demonstrating that auditory input contributes to balance regulation by enhancing spatial orientation and multisensory integration, particularly in older adults [10,11]. Clinical and observational studies indicate that the use of hearing aids can reduce the incidence of falls and improve postural stability, particularly among older adults with coexisting vestibular dysfunction [9]. However, some longitudinal research indicates that the benefits may depend on factors such as proper device fitting and user compliance [5].

Despite growing recognition of the auditory contribution to balance, most existing studies remain observational, and the mechanisms by which auditory rehabilitation influences postural regulation are still not fully understood. Importantly, to date, no research has systematically examined whether the addition of a neuromodulatory component integrated into hearing aids can provide measurable improvements in postural stability beyond standard amplification. To date, no research has systematically examined whether adding a neuromodulatory component integrated into hearing aids can produce measurable improvements in postural stability beyond standard amplification. This represents the specific knowledge gap addressed by the present study.

Photobiomodulation (PBM) has been investigated for its potential effects on neural activity, posture, and physiological processes, although available evidence remains preliminary [2]. Taopatch^®^ is a nanotechnology-based device designed to deliver localized PBM through light emission within therapeutic wavelengths activated by body heat. In the present study, this technology was adapted for integration into conventional hearing aids, providing a combined auditory and peripheral neurosensory intervention.

In the context of an aging population, understanding the relationship between auditory deficits and postural control is critical not only for developing effective fall prevention strategies but also for designing interdisciplinary rehabilitation programs. The novelty of this study lies in the application of Taopatch^®^ nanotechnology into conventional hearing aids, moving beyond traditional amplification to explore a combined auditory–neuromodulatory approach. This work aims to provide preliminary evidence on whether such integration can influence postural control in older adults with hearing loss.

## 2. Materials and Methods

### 2.1. Participants

The study involved 40 individuals with hearing loss, with a mean age of 77.28 ± 15.58 years. The sample included 19 males and 21 females. The relatively wide age variability reflects the real-world clinical population of hearing aid users and the epidemiological distribution of age-related hearing loss.

Participant recruitment was carried out by Audioclinik (Mori, Italy), a company specializing in hearing aid services. All subjects were already wearing hearing aids at the time of the visit, and all participants were fitted with behind-the-ear (BTE) hearing aids. Audiological records indicated a mean hearing loss of approximately 55 dB HL, consistent with moderate sensorineural hearing impairment. Participants had been using hearing aids for an average duration of about 5 years, indicating familiarity with amplified auditory input. The relatively high mean age of the participants reflects the typical epidemiological distribution of hearing loss, which is more prevalent among older adults. Age-related hearing loss (presbycusis) is, in fact, one of the most common chronic conditions affecting the elderly population [12].

Specific exclusion criteria were applied to ensure sample homogeneity and minimize potential confounding factors. Participants with known conditions affecting balance, such as neurological or vestibular disorders, were excluded. Additionally, individuals using walking aids or those who had consumed alcohol within the 24 h before the assessments were not included.

Written informed consent was obtained from all the participants, and they were told that they were free to withdraw from the study at any time without penalty. The study was approved by the Internal Research Board The study was approved by the Internal Research Board of the University of Enna “KORE” (No. 2574 of 8 February 2024). All procedures were carried out in accordance with the Declaration of Helsinki of the World Medical Association regarding the conduct of research.

Finally, although recruitment was carried out through a single clinic, this choice ensured homogeneity of procedures, instrumentation, and clinical expertise, thereby enhancing the reliability of the measurements and reducing variability associated with external factors.

### 2.2. Postural Evaluation

Each participant underwent postural evaluation across five separate sessions conducted under controlled laboratory conditions. During all sessions, participants wore their own hearing aids to ensure ecological validity and stability of the auditory input. A visually identical patch was affixed to the external surface of each hearing aid for every measurement session. However, no direct measurements of thermal or other biophysical properties were performed.

In four of these sessions, the patch was a placebo device, identical in appearance and weight to the active Taopatch^®^ but devoid of nanotechnological components. In the fifth session, the actual Taopatch^®^ was applied. Participants were not informed about when the active device was used to minimize suggestion effects and self-correction behaviors.

The first three placebo sessions were conducted to familiarize participants with the testing environment and were not included in the data analysis. The fourth session (placebo patch) served as the baseline condition, while the fifth session (Taopatch^®^) represented the experimental condition. The sessions were performed on different days to ensure reproducibility.

Measurements were performed using a baropodometric platform (Sensor Medica S.r.l., Guidonia Montecelio, Rome, Italy) integrated with Software Motux studio (version 1.10.14.0), which enabled the assessment of both stabilometric parameters, such as ellipse area, center of pressure (CoP), path length, and average CoP velocity, as well as static postural parameters. The latter included plantar pressure, load distribution between the lower limbs, and the support surface area of the feet.

Baropodometric stabilometry is a validated and widely used tool in clinical assessment of static postural control [13].

### 2.3. Treatment Procedure

The study involved experienced hearing aid users, ensuring that participants’ auditory conditions were stable and representative of their typical hearing performance.

In each evaluation, a patch was affixed to the external surface of the hearing aid shell, adjacent to the external auditory meatus, using medical-grade adhesive. No insertion into the ear canal was performed, guaranteeing participant safety and preserving the acoustic properties of the device.

As described above, four sessions employed a placebo patch, while the fifth used the Taopatch^®^ device. The patch type was not disclosed to participants at any time to prevent bias arising from psychological or expectation-related factors. The first three placebo sessions were used for familiarization and were excluded from the final analysis. The fourth session (placebo) constituted the control condition, and the fifth session (Taopatch^®^) represented the experimental condition. The experimental sessions followed a fixed sequence, with the placebo condition always preceding the active (Taopatch^®^) condition. Therefore, the study design should be considered a non-randomized within-subject pre–post comparison. This choice was made to ensure participant procedural consistency.

All patch applications were performed by a trained audiologist, who verified the stability of adhesion visually before each trial.

### 2.4. The Mechanism of Action

Taopatch^®^ is a nanotechnology-based medical device designed to promote health and well-being, based on current knowledge of biophysics applied to medicine. The device is classified as a nervous system stimulator, acting by potentially improving communication between the nervous system, muscles, and receptors through photobiomodulation (PBM) [14,15]. This classification does not imply a direct stimulation of specific neural structures, but rather a modulatory effect on neurophysiological processes, as described for other low-intensity PBM devices [14,16,17]. Taopatch^®^ is primarily activated by infrared radiation generated from body heat. Once activated, the photoselective nanomaterials contained in the device emit light across a broad spectrum of therapeutic wavelengths, with low intensity suitable for photobiomodulation.

Preliminary studies suggest that Taopatch^®^ may contribute to postural regulation [18], reduce pain [19], or influence vitamin D metabolism [20], although these effects remain hypotheses that require further experimental validation.

One of the main cellular responses to PBM is the potential activation of the mitochondrial enzyme cytochrome C-oxidase (CCO), which may increase ATP availability, supporting neuronal activity, synaptic communication, and muscle coordination [15]. Additional mechanisms include modulation of redox signaling and interaction with photosensitive ion channels, particularly transient receptor potential (TRP) channels, which are involved in neuronal excitability and sensory modulation [21,22]. Application of PBM adjacent to the external auditory meatus is hypothesized to allow modulation of vestibular and cochlear structures essential for postural control [23,24]. However, this hypothesis does not imply direct optical penetration to deep vestibular receptors, but rather an indirect influence on peripheral sensory inputs and central multisensory integration pathways. The vestibular system plays a central role in detecting head position and movement in space, continuously integrating with visual and proprioceptive inputs to maintain balance. Photonic stimulation in this area may promote improved multisensory integration; however, the precise neurofunctional mechanisms underlying this effect remain under investigation.

### 2.5. Statistical Analysis

Results are presented as mean ± standard deviation (SD). In addition to calculating the average percentage variations between conditions, paired t-tests were used to evaluate the statistical significance of the differences. Normality of all variables was assessed using the Shapiro–Wilk test, and all variables were found to be approximately normally distributed (*p* > 0.05), confirming the assumptions for the paired *t*-tests. Preliminary data screening also included checks for linearity, the presence of univariate and multivariate outliers, homogeneity of variance–covariance matrices, and absence of multicollinearity. Although these checks are not required for paired *t*-tests, they were performed to ensure overall data quality and the validity of the analyses; no violations were detected. To control for multiplicity, Bonferroni correction was applied to the primary outcomes (sway path length and mean center-of-pressure (CoP) velocity). Sway path length and mean center-of-pressure (CoP) velocity were defined as primary stabilometric outcomes, as they represent global indicators of postural stability. The remaining stabilometric and plantar pressure parameters were analyzed as secondary exploratory outcomes. Given their exploratory nature, secondary outcomes were not adjusted for multiple comparisons and were interpreted cautiously as hypothesis-generating. The effect size calculation (Cohen’s d) was used to evaluate the practical significance of findings, where values between 0 and 0.2 represent “very small” effects, values between 0.2 and 0.5 represent “small” effects, values between 0.5 and 0.8 represent “medium” effects, and values above 0.8 represent “large” effects. All tests were two-tailed. IBM SPSS 20.0 for Windows (SPSS, Inc., Chicago, IL, USA) was used for statistical analysis.

## 3. Results

### 3.1. Static Analysis

The static postural parameters analysis revealed significant changes in several postural parameters following the integration of Taopatch^®^ technology into the hearing aid’s earpiece, as reported in Table 1, which summarizes the statistical analysis of the static parameters.

**Figure 1:**
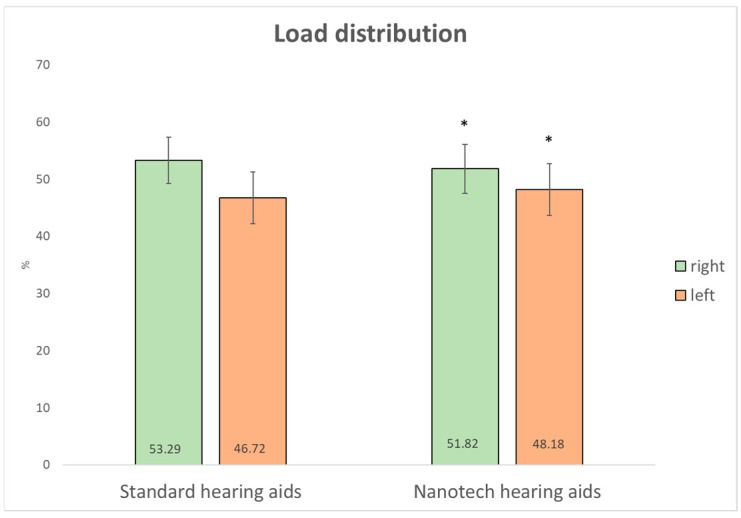
Load distribution with a standard hearing aid and with built-in Taopatch^®^ nanotechnology (* statistically significant).

In the open-eye condition, a lateral redistribution of body load was observed, with a significant reduction in load on the right foot (−3%, *p* = 0.01, d = 0.70) and a corresponding increase on the left foot (+3%, *p* = 0.01, d = 0.70). These results suggest not only statistical significance but also a meaningful effect on postural symmetry (Figure 1).

Moreover, both average and maximum pressures on the right foot decreased significantly (−3%, *p* = 0.002, d = 0.71 and −2%, *p* = 0.02, d = 0.61, respectively). These medium effects indicate a more homogeneous load distribution and a potential reduction in pressure peaks. A further confirmation of this is the 2% increase in the maximum pressure measured on the left foot.

**Figure 2:**
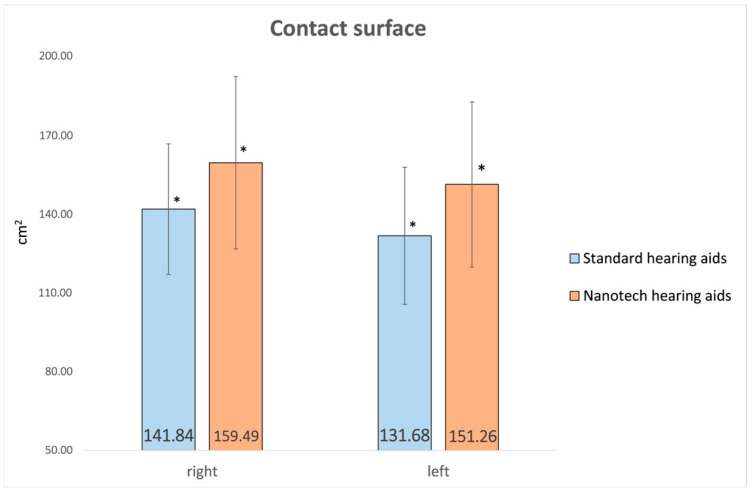
Comparison of Contact Surface (cm2) between Standard and Nanotechnological Hearing aids (* statistically significant).

The plantar contact surface showed a marked and statistically significant increase on both feet: +12% on the right foot (*p* < 0.001, d = 1.07) and +15% on the left foot (*p* < 0.001, d = 1.09), as illustrated in Figure 2. These large effect sizes suggest potentially meaningful differences in postural parameters beyond statistical significance alone. Although similar trends were observed in the eyes-closed condition, they did not reach statistical significance, suggesting that the effects may be more relevant in the presence of visual input.

### 3.2. Stabilometric Analysis

Improvements in multiple postural parameters were observed through stabilometric assessment after the inclusion of Taopatch^®^ technology within the hearing aid earpiece, as reported in Table 2, which summarizes the statistical analysis of the stabilometric parameters.

**Figure 3:**
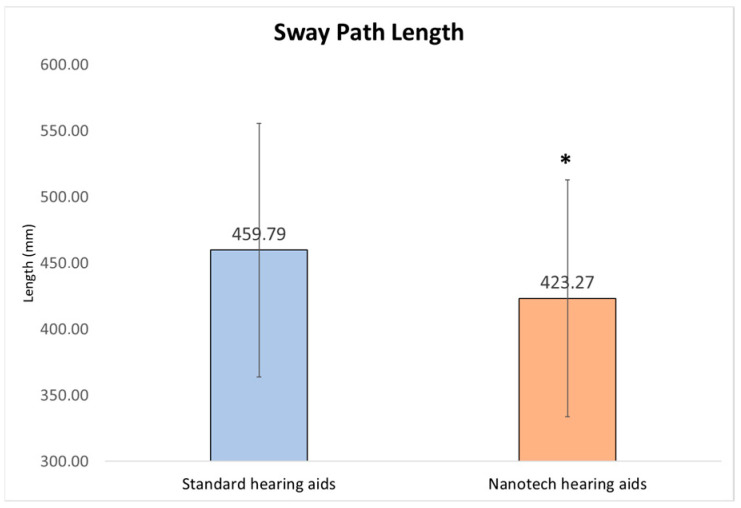
Comparison of Sway Path Length between Standard and Nanotechnological Hearing aids (* statistically significant).

Sway path length and mean center-of-pressure (CoP) velocity were defined as primary stabilometric outcomes, as they represent global indicators of postural stability. The remaining stabilometric and plantar pressure parameters were analyzed as secondary exploratory outcomes. With eyes open, a reduction in the sway path length (−8%, *p* = 0.002, d = 0.46) was observed, indicating decreased postural instability (Figure 3). There is a significant reduction in the sway path length, both along the horizontal axis (X Sway Path Length), with a decrease of 9% (*p* = 0.001, d = 0.66), and along the vertical axis (Y Sway Path Length), with a reduction of 6% (*p* = 0.05, d = 0.37). The decrease in mean velocity of the center of pressure (−8%, *p* = 0.002, d = 0.57) further supports these findings, indicating smoother postural adjustments. Similarly, speed variation decreased significantly (−14%, *p* = 0.02, d = 0.51) and mean acceleration was reduced (−15%, *p* = 0.02, d = 0.53). These medium-to-large effect sizes indicate statistically detectable differences and may reflect functional changes in postural regulation.

The LFS parameter (length as a function of area), calculated as the ratio between the total sway path length of the center of pressure (CoP) and the area of the confidence ellipse, captures the efficiency of postural control [25]. This parameter reflects the relationship between the spatial dispersion of CoP and the amount of corrective activity performed; lower values are generally interpreted as indicating more efficient postural regulation. The LFS showed a 30% reduction (*p* = 0.04, d = 0.49), suggesting greater postural stability at a lower energy cost. While the effect size remains in the small-to-medium range, the considerable percentage change highlights a clinically relevant improvement in postural efficiency, indicating a reduced energetic cost of maintaining balance.

In the eyes-closed condition, no statistically significant changes were observed. However, a trend toward postural stabilization was still apparent, indicating a limited effect of the device in the absence of visual input. These results suggest that even without visual input, the device promotes moderate enhancements in postural stability, though with smaller consistency compared to the open-eyes condition.

## 4. Discussion

### 4.1. Static Analysis

Postural stability declines with age due to reduced sensory input and structural brain changes [26]. In this study, the integration of Taopatch^®^ technology into the hearing aid earpiece was associated with measurable changes in static postural parameters, particularly in conditions with visual input; however, causality cannot be established. Accordingly, the results should be interpreted as exploratory and hypothesis-generating rather than as evidence of a definitive therapeutic effect. The results indicate trends toward a more balanced load distribution between feet and a broader plantar contact surface.

The effects were more evident under open-eye conditions, suggesting that the observed changes may be related to multisensory integration processes in which visual input interacts with auditory and somatosensory information. Under closed-eyes conditions, the reduced statistical significance of several parameters may indicate that any device-related modulation is insufficient to compensate for the absence of visual stabilization cues, which are known to play a dominant role in postural control in older adults.

A lateral redistribution of load was observed (slight shift from right to left foot), and the mean plantar pressure on the right foot decreased. These observations are descriptive and hypothesis-generating, and they may also reflect adaptive or compensatory postural strategies and normal physiological variability, which are common in elderly populations [27]. These parameters are clinically relevant, as asymmetries in foot load are associated with postural instability and increased musculoskeletal risk [28].

An increase in the plantar contact surface (+12% on the right and +15% on the left) was observed; this may indicate a broader base of support, but its physiological interpretation remains speculative. Increasing the contact surface in older people appears to be a compensatory strategy adopted by the postural system to improve balance [27] and should not be interpreted as evidence of improved proprioceptive integration. Although repeating the tests with eyes closed revealed similar trends, only some parameters reached statistical significance, while others did not. These findings suggest a potential influence of visual input, but they cannot confirm a causal effect of the device. This observation is consistent with Horak’s report that visual circuits contribute to the enhancement of proprioceptive and vestibular inputs, improving postural control [29].

In light of the evidence reported in the literature on photobiomodulation (PBM) and wearable photonic devices, the biological rationale underlying Taopatch^®^ does not rely on a local or direct interaction with the vestibular labyrinth, but rather on indirect systemic and neurophysiological mechanisms. Low-intensity PBM has been shown to modulate mitochondrial activity, particularly through the involvement of cytochrome c oxidase, leading to increased ATP production, modulation of reactive oxygen species, and activation of intracellular redox signaling pathways [30,31]. In addition, interactions with photosensitive ion channels (TRP channels) and membrane signaling mechanisms have been reported, supporting biological effects that may extend beyond the site of application [21,22].

Compared with conventional hearing aids, which act primarily through acoustic amplification, the present approach introduces a potential peripheral neurosensory modulation component. However, unlike galvanic vestibular stimulation or transcranial PBM, which more directly target central or vestibular pathways, the Taopatch^®^ system operates through indirect and low-intensity photonic stimulation at the peripheral level. Therefore, the magnitude and nature of its effects are likely to be subtler and more dependent on intact multisensory integration processes.

Previous research on vestibular stimulation (e.g., galvanic vestibular stimulation) or auriculotherapy provides context for potential mechanisms [32,33,34], but direct comparison is limited due to differences in intervention and population. Consequently, the effects observed on balance control in the present study may be interpreted as a functional modulation of central sensory integration systems—proprioceptive, vestibular, and visual—rather than as a mechanical or optical direct action on the deep vestibular apparatus, in agreement with previous reports on PBM and non-invasive sensory stimulation devices [35,36].

### 4.2. Stabilometric Analysis

The results show observable trends toward improvement in postural parameters following the application of the acoustic devices. In particular, the sway path length showed an average reduction of 8% (*p* = 0.002), suggesting an overall decrease in corrective postural activity. This indicator, often used as a global measure of postural stability, reflects the amount of movement made by the center of pressure (CoP) during the test. Its reduction may indicate a tendency toward improved ability to maintain a stable upright position.

Analyzing the directional components, a more pronounced reduction was observed along the mid-lateral axis (X sway path length: −9%, *p* = 0.001) than in the anteroposterior axis (Y sway path length: −6%, *p* = 0.05). These observations suggest possible greater effects of the device on lateral sway, which is known to be more difficult for the postural system to control [37]. From a postural control perspective, medio-lateral sway is considered more sensitive to balance impairment than anteroposterior sway, as lateral stability relies more heavily on hip-mediated strategies and sensory integration processes. Therefore, the preferential reduction in medio-lateral oscillations observed in this study may reflect a functional change in postural regulation strategies rather than a generalized reduction in movement amplitude. Under static postural control, a reduction in medio-lateral oscillations could reflect improved postural strategies, although no causal inference can be made. Given that quiet standing is predominantly governed by automatic postural control mechanisms and that participants were unaware of the timing of data acquisition, the likelihood that these changes are attributable to a learning or habituation effect appears limited. The average speed of CoP decreased by 8% (*p* = 0.002), with speed variation reduced by 14% (*p* = 0.02) and average acceleration by 15% (*p* = 0.01). These results suggest a general trend toward stabilization of posture, potentially reflecting more efficient corrective adjustments. Still, mechanistic explanations remain speculative [38,39]. The only parameter that did not show a statistically significant change was the ellipse surface, despite a decreasing trend. A possible explanation lies in the high heterogeneity of the sample, which included both elderly subjects with high surface areas (typically associated with greater instability) and subjects with extremely low values, consistent with excessively rigid and hyper-controlled postural control. This variability probably increased the standard deviation of the parameter, reducing its statistical power. The LFS showed a significant reduction of 30% (*p* = 0.05). Although the absolute value of LFS in our sample was higher than in the European reference study on the healthy population [40], its significant reduction confirms an improvement in the consistency between corrective activity and the spatial distribution of postural movement, although this finding should be interpreted cautiously, given the exploratory nature of the study.

Under closed-eye conditions, only the Y sway path length showed a significant variation, suggesting that stabilization trends were partially preserved without visual input. While previous studies suggest that sensory stimulation can influence postural control, the present results cannot establish direct neurophysiological mechanisms [2,3,14,15]. This pattern reinforces the interpretation that visual information plays a key role in the observed effects and that the device-related modulation, if present, likely operates within a multisensory framework rather than through isolated proprioceptive or vestibular enhancement.

Overall, the results suggest that Taopatch^®^-integrated acoustic devices may influence postural parameters under specific conditions, potentially through multisensory integration mechanisms. However, no definitive conclusions can be drawn regarding the modulation of neural pathways or proprioceptive integration, and the findings should be interpreted as descriptive and hypothesis-generating [41]. Placebo effects and physiological variability remain possible contributing factors and are therefore acknowledged as relevant limitations.

Previous studies on transcranial photobiomodulation (tPBM) and other sensory stimulations have reported temporary improvements in balance; however, direct mechanistic parallels to our research cannot be confirmed [42]. Similarly, auricular or cervical vibrotactile stimulation has been associated with potential postural benefits, but direct extrapolation is limited [3].

In this context, the Taopatch^®^ ear device could act through similar mechanisms, stimulating microcirculation and nerve receptors in the auricular skin, thereby promoting more effective control of postural oscillations [43].

Notably, individuals with moderate hearing loss exhibit decreased postural control, underscoring the functional connection between auditory deficits and balance regulation [4]. This supports the rationale for integrating auditory and neurosensory interventions; however, larger randomized, sham-controlled trials are required to substantiate the clinical impact.

## 5. Conclusions

The results obtained from this investigation suggest preliminary trends that indicate that the integration of Taopatch^®^ technology within the hearing aid earpiece was associated with changes in static and stabilometric postural parameters. Observed trends included reduced sway path length, lower CoP velocity, and modifications in plantar load distribution, indicating a possible shift toward more stable postural behavior under the tested conditions; however, these findings should be interpreted cautiously due to the limited sample size and study design. Although some parameters showed medium-to-large effect sizes according to conventional statistical benchmarks, effect size magnitude alone does not establish clinical relevance. Therefore, these differences should be interpreted as potentially meaningful functional variations rather than as confirmed clinically significant improvements. These effects appeared more evident in conditions with visual input, indicating that the observed changes were influenced by sensory context and may depend on the presence of visual stabilization cues; however, the present data do not allow conclusions regarding specific neural or vestibular mechanisms, and causal mechanisms cannot be confirmed. Overall, the study provides preliminary evidence that combining auditory amplification with a wearable photobiomodulation-based component may be associated with short-term modifications in postural control parameters. The hypothesis that photobiomodulation-based wearable technologies may influence balance-related functional outcomes should therefore be considered speculative and warrants further investigation, particularly in larger, randomized studies and in clinical or vulnerable populations.

Future research should employ larger samples, randomized sham-controlled designs, and objective neurophysiological or imaging measures to clarify the reproducibility, underlying mechanisms, and the actual clinical relevance of these observations.

### Limitations

The present study has several limitations that should be considered when interpreting the results. The sample size was relatively small, including 40 participants with considerable age variability (77.3 ± 15.6 years), which may limit the generalizability of the findings and increase the potential for bias. The study was also conducted in a single clinical setting, which may limit the representativeness of the sample and the generalizability of the findings to other populations or contexts. Furthermore, because the active condition was always administered after the placebo condition, a residual order effect cannot be entirely excluded. Although static postural control during quiet standing is generally considered to be minimally influenced by learning or practice effects and familiarization sessions were conducted to further reduce this possibility, the fixed sequence of administration may still have contributed, at least in part, to the observed changes. Future studies should implement randomized or counterbalanced designs to more rigorously control for potential order effects. Longitudinal studies examining the sustained impact of the device over time would also be valuable, as would exploring its effectiveness across diverse populations and clinical settings. Finally, the present investigation focused exclusively on older adults with hearing loss. Other populations, such as children with cochlear implants or pediatric hearing aid users, are known to exhibit a higher prevalence of balance disorders and may represent an important target for future studies aimed at exploring the potential role of nanotechnology-based or neurosensory interventions in balance control.

## Figures and Tables

**Table 1:** Results of static analysis (* statistically significant; **^§^** borderline).

	Open Eyes									Closed Eyes								
	Standard			Nanotech			∆ Diff %	*p*	Cohen’s d	Standard			Nanotech			∆ Diff %	*p*	Cohen’s d
Lateral distribution (%)																		
right	53.29	±	4.0	51.82	±	4.3	−3%	0.01 *	0.70	52.58	±	4.5	52.06	±	4.5	−1%	0.12	0.21
left	46.72	±	4.0	48.18	±	4.3	3%	0.01 *	0.70	47.43	±	4.5	47.94	±	4.5	1%	0.12	0.19
Front/back distribution (%)																		
front	49.08	±	5.5	48.63	±	5.4	−1%	0.39	0.11	48.70	±	6.4	48.13	±	4.9	−1%	0.21	0.15
back	50.93	±	5.5	51.13	±	5.1	0%	0.26	0.14	51.30	±	6.4	51.88	±	4.9	1%	0.21	0.16
Average pressures (KPa)																		
right	129.00	±	8.1	125.07	±	8.6	−3%	0.002 *	0.71	127.85	±	8.4	125.37	±	9.0	−2%	0.05 ^§^	0.59
left	121.52	±	9.3	120.24	±	8.4	−1%	0.38	0.13	121.44	±	9.8	121.48	±	8.4	0%	0.37	0.21
Maximum pressures (KPa)																		
right	243.42	±	10.7	238.71	±	13.6	−2%	0.02 *	0.61	240.46	±	13.9	238.03	±	13.5	−1%	0.18	0.20
left	230.41	±	20.6	235.36	±	21.1	2%	0.02 *	0.65	232.11	±	20.7	236.71	±	20.7	2%	0.01 *	1.09
Contact surface (cm^2^)																		
right	141.84	±	24.8	159.49	±	32.8	12%	<0.001 *	1.07	144.70	±	30.1	159.28	±	33.9	10%	<0.001 *	1.08
left	131.68	±	26.1	151.26	±	31.4	15%	<0.001 *	1.09	137.08	±	30.8	150.61	±	31.5	10%	<0.001 *	1.09
Forefoot load (%)																		
right	27.26	±	4.1	26.83	±	3.7	−2%	0.13	0.11	26.70	±	4.8	26.16	±	4.1	−2%	0.20	0.09
left	22.10	±	3.0	22.81	±	2.8	3%	0.07	0.13	22.13	±	3.2	22.32	±	2.5	1%	0.19	0.12
Backfoot load (%)																		
right	26.13	±	3.8	25.49	±	3.3	−2%	0.09	0.13	26.04	±	4.1	25.99	±	3.6	0%	0.30	0.25
left	24.45	±	3.6	24.89	±	2.9	2%	0.04 *	0.44	25.14	±	4.2	25.52	±	3.6	2%	0.28	0.21

**Table 2:** Results of Stabilometric analysis (* statistically significant; ^§^ borderline).

		Open Eyes									Closed Eyes								
		Standard			Nanotech			∆ Diff %	*p*	Cohen’s d	Standard			Nanotech			∆ Diff %	*p*	Cohen’s d
Ellipse surface	mm^2^	37.6	±	36.3	36.3	±	24.3	−4%	0.41	0.27	81.9	±	76.2	77.0	±	105.1	−6%	0.38	0.25
Sway Path Length	mm	459.8	±	95.8	423.3	±	89.6	−8%	0.002 *	0.46	529.7	±	149.5	496.8	±	148.0	−6%	0.05 ^§^	0.35
X Sway Path Length	mm	336.0	±	75.2	304.8	±	56.7	−9%	0.001 *	0.66	368.3	±	92.9	354.0	±	91.2	−4%	0.14	0.17
Y Sway Path Length	mm	249.4	±	55.6	235.2	±	66.7	−6%	0.05 ^§^	0.37	307.4	±	107.4	279.5	±	107.1	−9%	0.03 *	0.61
Mean Velocity	mm/s	9.0	±	1.9	8.3	±	1.7	−8%	0.002 *	0.57	10.5	±	3.0	9.7	±	2.9	−7%	0.05 ^§^	0.47
Speed Variation	mm/s	35.3	±	16.0	30.3	±	14.2	−14%	0.02 *	0.51	57.7	±	47.2	60.9	±	83.9	6%	0.40	0.16
Mean Acceleration	mm/s	0.7	±	0.3	0.6	±	0.3	−15%	0.02 *	0.53	1.1	±	0.9	1.2	±	1.6	6%	0.39	0.19
COP (Center of Pressure)																			
X COP	mm	−4.6	±	6.8	−4.0	±	5.9	−12%	0.25	0.17	−3.7	±	7.7	−3.2	±	5.9	−14%	0.31	0.19
Y COP	mm	−14.6	±	6.6	−14.9	±	6.0	2%	0.30	0.21	−15.1	±	7.9	−15.4	±	6.4	2%	0.31	0.15
LFS (Low Frequency Sway)	mm^2^	24.2	±	24.1	17.0	±	10.8	−30%	0.04 *	0.49	13.1	±	14.9	15.5	±	14.6	18%	0.21	0.18
MP (Medium Power) peak amplitude	mm	789.3	±	446.8	711.0	±	361.7	−10%	0.15	0.17	471.5	±	492.6	563.7	±	478.7	20%	0.12	0.21
SP (Spectral Power) peak variability	mm	316.2	±	153.2	290.0	±	137.4	−8%	0.20	0.19	204.1	±	155.1	240.6	±	172.5	18%	0.08	0.29

## Data Availability

The raw data supporting the conclusions of this article will be made available by the authors upon request.
